# Effect and safety of acupuncture on cerebrovascular reserve in patients with acute cerebral infarction: A protocol for systematic review and meta-analysis

**DOI:** 10.1097/MD.0000000000026636

**Published:** 2021-07-16

**Authors:** Yuliang Wang, Jijie Xing, Ying Li, Rui Zhang

**Affiliations:** a Department of Rehabilitation, Beijing Xiaotangshan Hospital, Beijing, China; b Department of Acupuncture, The Second Affiliated Hospital of Heilongjiang University of Chinese Medicine, Heilongjiang, China; c Department of Science and Education, Beijing Xiaotangshan Hospital, Beijing, China; d Department of Rehabilitation, Hotan District People's Hospital, Xinjiang, China.

**Keywords:** acupuncture, acute cerebral infarction, meta-analysis, protocol, systematic review

## Abstract

**Background:**

As far as we know, no evidence has been established to assess the effects of acupuncture for acute cerebral infarction patients. Therefore, this systematic review and meta-analysis will be conducted to assess the efficacy and safety of acupuncture on cerebrovascular reserve in patients with acute cerebral infarction.

**Methods:**

On June 20, 2021, the authors will perform a preliminary search in the PubMed, Embase, and Scopus databases using the following keywords: “acupuncture,” “acute cerebral infarction.” We will also examine the Clinical Trials Registry for other ongoing and unpublished studies. The inclusion criteria included (1) patients with acute cerebral infarction, (2) patients who received acupuncture, and (3) studies assessed cerebrovascular reserve, breath-holding index, Barthel index, and adverse events. All English language randomized controlled trials published within the last 20 years were eligible for inclusion. Primary outcome measures in our study are cerebrovascular reserve, and secondary outcome measures include the breath-holding index, Barthel index, and adverse events. All outcomes are pooled on a random-effect model.

**Results:**

The results of this research will be delivered in a peer-reviewed journal.

**OSF registration number:**

10.17605/OSF.IO/7M4BK.

## 1 Introduction

Acute cerebral infarction remains a global concern because its incidence is highest in Asia, particularly in China, and Eastern Europe. Moreover, according to the European Stroke Burden Report, if there is no change in the incidence of cerebral infarction, and the total number of stroke events will increase by more than 30% between 2015 and 2035.^[1,2]^ Although early diagnosis and management can usually reverse most acute neurological defects, the prognosis of acute cerebral infarction can be devastating, and severe irreversible neurological consequences can affect the patient's physical and mental health. Furthermore, follow-up long-term care is a huge burden on socio-economic development.^[3]^

Cerebral infarction is closely related to cerebral vascular stenosis or cerebral thrombosis. When the cerebral vascular reserve can no longer support the further increase of cerebral perfusion, the brain tissue can only maintain the intracranial blood supply by increasing the oxygen fraction extraction.^[4]^ However, as the degree of cerebral hypoxia worsens, the occurrence of acute infarction becomes more likely. Patients with low cerebrovascular reserve tend to experience more severe hypoperfusion, ischemia, and hypoxia, leading to infarction and further tissue damage. Insufficient cerebral vascular reserve is not only an independent risk factor for cerebral infarction but also a risk factor for the progression and recurrence of cerebral infarction.^[5,6]^ Therefore, it is very important to develop new strategies to increase cerebral vascular reserve in patients with acute infarction to prevent the progression and recurrence of infarction.

Acupuncture is considered to be a promising complementary and comprehensive approach to lowering blood pressure without many of the limitations of medical interventions. It is a practice in traditional East Asian medicine in which specific points on the body are stimulated, most commonly by inserting a disposable fine stainless steel needle into the skin. Acupuncture has been shown to relieve various types of pain and conditions, including cardiovascular disease.^[7,8]^ Western culture is increasingly embracing acupuncture. The American College of Physicians’ 2017 clinical practice guidelines list acupuncture as a non-drug treatment option for acute and chronic low back pain.^[9]^ However, the effects of acupuncture on the cardiovascular system are still poorly studied, which hampers its use as a treatment option in the Western world. As far as we know, no evidence has been established to assess the effects of acupuncture for acute cerebral infarction patients. Therefore, this systematic review and meta-analysis will be conducted to assess the efficacy and safety of acupuncture on cerebrovascular reserve in patients with acute cerebral infarction.

## 2 Materials and methods

### 2.1 Information sources and search strategy

The protocol was written in accordance with the Preferred Reporting Items for Systematic Reviews and Meta-Analyses Protocols statement guidelines. Ethical approval is not required for our study as all analyses will be based on pooled data from previously published studies. On June 20, 2021, the authors will perform a preliminary search in the PubMed, Embase, and Scopus databases using the following keywords: “acupuncture,” “acute cerebral infarction.” We will also examine the Clinical Trials Registry for other ongoing and unpublished studies. In addition, we will search for references to the most relevant articles. The prospective registration has been approved by the Open Science Framework Registry (https://osf.io/7m4bk) under registration number 10.17605/OSF.IO/7M4BK.

### 2.2 Inclusion and exclusion criteria

The inclusion criteria included (1) patients with acute cerebral infarction, (2) patients who received acupuncture, and (3) studies assessed cerebrovascular reserve, breath-holding index, Barthel index, and adverse events. All English language randomized controlled trials published within the last 20 years were eligible for inclusion. The exclusion criteria were as follows: (1) studies that did not assess the above outcomes; (2) no direct comparison of acupuncture and other treatment; and (3) studies with the following types: case reports, comments or letters, biochemical trials, protocols, conference abstracts, and reviews.

### 2.3 Study selection

The first author will conduct a preliminary screening based on the title to eliminate any research not related to the topic. A log of excluded studies is kept with the rationale for exclusion. Subsequently, all remaining abstracts will be reviewed by the primary author, and the selection criteria are applied. Studies identified for full-text review will be evaluated by 2 authors for inclusion in the study. Disagreements will be resolved through a discussion with a third review author. Journal titles and author's names will be not glossed over in the research selection process. A manual search of the bibliographies of included studies is performed to ensure that the overall search was comprehensive and complete.

### 2.4 Assessment of study quality

We will assess the quality of the included randomized controlled trials using the Oxford Quality Scoring System, which evaluates studies based on randomization, blinding, and a description of withdrawals and dropouts. Interobserver differences in assessing quality or incongruity will be resolved by consensus between authors.

### 2.5 Outcome measures

Primary outcome measures in our study are cerebrovascular reserve, and secondary outcome measures include breath-holding index, Barthel index, and adverse events. Adverse events are defined as negative or unintended clinical manifestations following the treatment.

### 2.6 Statistical analysis

Review Manager software (v 5.3; Cochrane Collaboration) is used for the meta-analysis. Extracted data are entered into Review Manager by the first independent author and checked by the second independent author. Risk ratio with a 95% confidence interval or standardized mean difference with 95% CI is assessed for dichotomous outcomes or continuous outcomes, respectively. The heterogeneity is assessed using the *Q* test and *I*^2^ statistic. An *I*^2^ value of <25% is chosen to represent low heterogeneity and an *I*^2^ value of >75% to indicate high heterogeneity. All outcomes are pooled on a random-effect model. A *P* value of <.05 is considered to be statistically significant.

## 3 Discussion

Acupuncture is considered to be a promising complementary and comprehensive approach to lowering blood pressure without many of the limitations of medical interventions. It is a practice in traditional East Asian medicine in which specific points on the body are stimulated, most commonly by inserting a disposable fine stainless steel needle into the skin. Acupuncture has been shown to relieve various types of pain and conditions, including cardiovascular disease.^[7,8]^ Western culture is increasingly embracing acupuncture. The American College of Physicians’ 2017 clinical practice guidelines list acupuncture as a non-drug treatment option for acute and chronic low back pain.^[9]^ However, the effects of acupuncture on the cardiovascular system are still poorly studied, which hampers its use as a treatment option in the Western world. As far as we know, no evidence has been established to assess the effects of acupuncture for acute cerebral infarction patients. Therefore, this systematic review and meta-analysis will be conducted to assess the efficacy and safety of acupuncture on cerebrovascular reserve in patients with acute cerebral infarction.

## Author contributions

**Conceptualization:** Ying Li, Rui Zhang.

**Data curation:** Yuliang Wang, Jijie Xing.

**Formal analysis:** Yuliang Wang, Jijie Xing.

**Investigation:** Yuliang Wang, Jijie Xing.

**Methodology:** Ying Li.

**Project administration:** Rui Zhang.

**Resources:** Ying Li, Rui Zhang.

**Software:** Rui Zhang, Ying Li.

**Supervision:** Jian Huang, Rui Zhang.

**Validation:** Jijie Xing.

**Visualization:** Jijie Xing.

**Writing – original draft:** Yuliang Wang.

**Writing – review & editing:** Rui Zhang.
